# The Identification of Proteoglycans and Glycosaminoglycans in Archaeological Human Bones and Teeth

**DOI:** 10.1371/journal.pone.0131105

**Published:** 2015-06-24

**Authors:** Yvette M. Coulson-Thomas, Vivien J. Coulson-Thomas, Andrew L. Norton, Tarsis F. Gesteira, Renan P. Cavalheiro, Maria Cecília Z. Meneghetti, João R. Martins, Ronald A. Dixon, Helena B. Nader

**Affiliations:** 1 Department of Biochemistry, Universidade Federal de São Paulo, São Paulo, Brazil; 2 School of Life Sciences, University of Lincoln, Lincoln, Lincs, United Kingdom; 3 Department of Ophthalmology, College of Medicine, Edith J. Crawley Vision Research Center, University of Cincinnati, Cincinnati, Ohio, United States of America; 4 Durham University, Durham, United Kingdom; University of Insubria, ITALY

## Abstract

Bone tissue is mineralized dense connective tissue consisting mainly of a mineral component (hydroxyapatite) and an organic matrix comprised of collagens, non-collagenous proteins and proteoglycans (PGs). Extracellular matrix proteins and PGs bind tightly to hydroxyapatite which would protect these molecules from the destructive effects of temperature and chemical agents after death. DNA and proteins have been successfully extracted from archaeological skeletons from which valuable information has been obtained; however, to date neither PGs nor glycosaminoglycan (GAG) chains have been studied in archaeological skeletons. PGs and GAGs play a major role in bone morphogenesis, homeostasis and degenerative bone disease. The ability to isolate and characterize PG and GAG content from archaeological skeletons would unveil valuable paleontological information. We therefore optimized methods for the extraction of both PGs and GAGs from archaeological human skeletons. PGs and GAGs were successfully extracted from both archaeological human bones and teeth, and characterized by their electrophoretic mobility in agarose gel, degradation by specific enzymes and HPLC. The GAG populations isolated were chondroitin sulfate (CS) and hyaluronic acid (HA). In addition, a CSPG was detected. The localization of CS, HA, three small leucine rich PGs (biglycan, decorin and fibromodulin) and glypican was analyzed in archaeological human bone slices. Staining patterns were different for juvenile and adult bones, whilst adolescent bones had a similar staining pattern to adult bones. The finding that significant quantities of PGs and GAGs persist in archaeological bones and teeth opens novel venues for the field of Paleontology.

## Introduction

Bone tissue is mineralized dense connective tissue that consists mainly of a mineral component (hydroxyapatite) and an organic matrix. The organic matrix is comprised mainly of collagen types I and III, non-collagenous proteins such as fibronectin, osteocalcin, osteopontin, osteonectin and bone sialoprotein II, and proteoglycans (PGs) such as decorin and biglycan [1,2]. Extracellular matrix (ECM) proteins bind very tightly to hydroxyapatite which protects these proteins from the destructive effects of temperature and chemical agents after death [3]. PGs and their constituent glycosaminoglycan (GAG) chains also bind to hydroxyapatite [4,5], which could also potentially protect these molecules from degradation. ECM proteins, such as collagen type I and osteonectin, have been successfully extracted from archaeological skeletons [3,6,7], and hexosamine has been identified in fossilized cartilage [8]; however, there is a lack of research concerning PGs and GAG chains in archaeological bones and teeth, even though these molecules play a major role in bone morphogenesis, homeostasis and degenerative bone disease [9,10].

PGs are macromolecules comprised of one or more GAG chains covalently bound to a protein core [11]. GAGs are linear polysaccharides composed of repeating disaccharide units which consist of a hexosamine and either hexuronic acid or galactose units, and may be sulfated in various positions. The strategic variability in sulfate substitution results in considerable sequence heterogeneity which conveys the plethora of biological roles GAGs play in cell-cell and cell-matrix interactions. GAGs are classified into six groups: chondroitin 4- and 6-sulfate (C4S, C6S), keratan sulfate (KS), hyaluronic acid (HA), dermatan sulfate (DS), heparin and heparan sulfate (HS).

Small Leucine Rich Proteoglycans (SLRPs) are an important family of PGs described in bones. SLRPs are localized in most skeletal regions and play a major role during all phases of bone formation, including cell proliferation, organic matrix deposition, remodeling, and mineral deposition. Biglycan and decorin are two such SLRPs described in bones, which have CS/DS GAG substitutions. Decorin expression begins at early bone matrix deposition, whilst biglycan is expressed during cell proliferation, ceases to be expressed during the period of bone matrix deposition, and is once again expressed during mineralization [12]. Fibromodulin, lumican, keratocan, proline/arginine-rich end leucine-rich repeat protein (PRELP), osteoadherin and osteoglycin/mimecan are SLRPs described in bone that may have KS chains or polylactosamine side chains. Lumican and osteoglycin/mimecan have been described in avian medullary bone [13]. Lumican has also been shown to be secreted by differentiating and mature murine osteoblasts [14]. Osteoadherin has been described in bovine mineralized bone matrix and is secreted by osteoblasts [15]. PRELP has been described in conjunctive tissue and impairs osteoclastogenesis and bone resorption [16]. Fibromodulin has been shown to be expressed in murine chondrocytes and osteoblasts during endochondral and intramembranous ossification [17]. Keratocan is expressed by osteoblasts modulating osteoblast function, and keratocan knockout mice have decreased rates of bone formation and mineral deposition [18]. In teeth, biglycan, decorin, fibromodulin and lumican are the predominant PGs of predentin and dentin and play a role in dentinogenesis [19]. In addition, biglycan, decorin, fibromodulin and lumican have been described in cementum [20,21].

SLRPs regulate collagen fibrillogenesis via their bi-functional character; the protein moiety interacts with the collagen fibrils and the GAG chains regulate interfibrillar distances [22,23,24,25,26]. In addition, SLRP binding to collagen has been shown to enhance collagen fibril stability [27,28] and to protect collagen fibrils from proteolytic cleavage by various collagenases [29]. SLRPs also bind growth factors; for example, biglycan, decorin and fibromodulin bind TGF-β in the extracellular matrix [30].

Given the vital role SLRPs and GAGs play in bone morphogenesis and homeostasis, alterations in their expression profile or their loss due to unregulated proteolysis leads to a plethora of diseases. The expression of PGs and GAGs is altered in various bone related diseases, including osteoarthritis of the temporomandibular joint [31], osteochondromas and peripheral chondrosarcomas [32], and due to this, these molecules can be used as biological markers.

This is the first time to our knowledge that PGs and GAGs have been analyzed in archaeological human bones and teeth. Initially, methods were optimized for the extraction of PGs and GAGs from archaeological human bones and teeth. Following extraction, the PGs and GAGs were characterized, and subsequently immunolocalized in archaeological human bone slices. The analysis of PGs and GAGs in archaeological skeletons is novel and could prove to be extremely useful in the fields of Paleontology and Forensic Science.

## Materials and Methods

### Samples

The bones and teeth used in this study were provided by the University of Lincoln, Lincoln, UK. Sixty-eight partial or complete skeletal remains were excavated in the last decade from a site on the south side of Monk’s Road, Lincoln (Ref. SK980714). The site was determined to be the extramural graveyard of the defunct parish of St Peter at Welles (*ad fontem*). The age of the site was estimated to 1150–1400 AD. The samples used in this study were from various types of archaeological human bone (femur, humerus, radius, tibia and ulna), identified as juvenile, adolescent or adult, and teeth, which are numbered from 1 to 32 and stored in the repository labeled LMK 03 076 in the secure laboratories of the School of Life Sciences, University of Lincoln, Brayford Pool LN6 7TS, UK.

### Ethics statement

All necessary permits were obtained for the described study, which complied with all relevant regulations. The appropriate Coroners license was obtained to excavate the skeletons and the research was approved by the University of Lincoln ethics committee in the UK and CEP/UNIFESP in Brazil (CAAE: 07934412.2.0000.5505).

### Bone and tooth preparation

The bones and teeth were initially cleaned in water and allowed to dry. In addition, bone samples were mechanically cleaned with autoclaved sandpaper. Subsequently, the bone and tooth samples were exposed to sodium hypochlorite for 15 min at room temperature. Bone powder and tooth powder were collected using a drill (Draper) with drill bits that had been previously exposed to sodium hypochlorite and autoclaved. The bone powder and tooth powder were stored at -20°C until processed. Face masks, gloves and clean laboratory coats were used when handling the skeletal material and carrying out procedures.

### Proteoglycan extraction

Bone and tooth powders were obtained as described above and suspended in 4 M guanidine-HCl, 20 mM NaH_2_PO_4_, 30 mM Na_2_HPO_4_, 300 mM EDTA, pH 7.4 (containing complete proteinase inhibitor cocktail, Roche, Basel, Switzerland) (25–100 mg/4.5 mL) and maintained under constant rotation at 4°C for 24 hours. Following the incubation period, double the volume was added of 7 M urea, 0.3 M NaCl, 0.05 M CH_3_COONa, pH 6.5 (containing complete proteinase inhibitor cocktail, Roche). The solution was then maintained under constant rotation at 4°C for an additional 24 hours, filtered through a Poly-Prep Chromatography Column (Bio-Rad, Hercules, CA), concentrated and desalinized using an Amicon Ultra-15 centrifugal filter device (Millipore, County Cork, Ireland) and vacuum dried. The crude protein/PG extract was then suspended (1 μg/μL) in MilliQ water containing complete proteinase inhibitor cocktail (Roche) and the PGs analyzed by agarose gel electrophoresis.

### Glycosaminoglycan extraction

GAGs were extracted according to a previously described method [33] with a few modifications. Bone and tooth powders were obtained as described above and suspended (25–50 mg/mL) in solution containing the proteolytic enzyme maxatase (Biocon Laboratories, São Paulo, Brazil) (4 mg/mL) in 0.05 M Tris-HCl buffer, pH 8.0, containing 1 M NaCl and incubated at 60°C for 2 days. A 1:10 volume of trichloroacetic acid was added and the samples kept on ice for 15 min. The samples were then centrifuged (15 min, 2250 g), the supernatant collected and the GAGs precipitated by slowly adding 2 volumes of methanol whilst vortexing and maintaining at -20°C overnight followed by centrifugation (10 min, 2250 g). The pellet was dried and suspended in MilliQ water (1 μg/μL).

### Agarose gel electrophoresis

PGs and GAGs were analyzed by 0.6% agarose gel electrophoresis in 0.05 M propanediamine acetate (PDA) buffer, pH 9, as previously described [34]. In this agarose gel electrophoresis system, GAGs and PGs migrate through the gel according to their affinity with the PDA buffer, which is dictated by the fine structure of the GAG chain. Following electrophoresis, the gels were submerged in 0.2% CETAVLON (cetyltrimethylammonium bromide, Sigma-Aldrich, St. Louis, MO) for 1 hour at room temperature, which precipitates tetrasaccharides and larger GAG fragments, and then the gels were dried. In the case of PG analysis, the gels were first submerged in formol:methanol (1:4 v/v) for 30 min and then in the CETAVLON. PG gels were stained by amido black (for staining proteins) and then stained for 30 min with 0.1% toluidine blue prepared in a solution of 1% acetic acid, 50% ethanol and 49% water, and destained with the same solution without toluidine blue (for staining CSPGs, DSPGs and HSPGs). As a result of this sequential staining process, the protein core is stained blue and the GAG chains are stained purple. GAG gels were stained with 0.1% toluidine blue prepared in a solution of 1% acetic acid, 50% ethanol and 49% water, destained with the same solution without toluidine blue (for staining CS, DS and HS), and then restained with 0.1% toluidine blue prepared in 25 mM sodium acetate buffer, pH 5.0, and destained in this solution without toluidine blue (for also staining HA). Gels were scanned and quantified using a densitometer and the Quick Scan 2000 program (Helena Laboratories, TX, USA), using CS, DS and HS (extracted from shark cartilage; all 1 mg/mL) applied to the same gels as standards.

### Digestion of glycosaminoglycans with chondroitinases AC and ABC

GAGs (60 μg) extracted from archaeological human bone samples were suspended in MilliQ water (60 μL) and the sample divided in three, boiled for 15 min to inactivate any residual maxatase, and lyophilized. The first sample was suspended in 20 μL chondroitin AC lyase (1 U/mL, Sigma-Aldrich, St. Louis, MO), the second sample in 20 μL chondroitin ABC lyase (1 U/mL, Sigma-Aldrich) and the third sample in 20 μL MilliQ water. The samples were incubated overnight at 37°C and 5 μL of each sample analyzed by agarose gel electrophoresis as mentioned above. Digestion control samples (C4S and a mixture of CS, DS and HS extracted from shark cartilage; all 1 mg/mL) were also incubated in chondroitin AC lyase, chondroitin ABC lyase or MilliQ water overnight at 37°C and analyzed by agarose gel electrophoresis.

### Digestion of glycosaminoglycans with heparin lyase II and a Flavobacterium heparinum extract

GAGs (60 μg) extracted from archaeological human bone samples were subjected to β-elimination to ensure that the GAGs (other than N-linked KS chains) had been released from the protein core. Briefly, the GAG pellet was suspended in 200 μL sodium borohydride (0.0378 g Na(BH_4_) in 1 mL NaOH 0.1 M) and kept overnight at room temperature. The solution was then neutralized using 10% acetic acid (approximately 110 μL). A volume of 500 μL of MilliQ water was added and the solution transferred to 1 kDa dialysis tubes (GE Healthcare Bio-Sciences Corp., NJ, USA), and dialyzed at room temperature for 4 hours in distilled water. The solution was then lyophilized. The GAG pellet was suspended in 60 μL MilliQ water and the sample divided in three, boiled for 15 min to inactivate any residual maxatase, and lyophilized. The first sample was suspended in 2.5 μL heparin lyase II (1 U/mL, Sigma-Aldrich, St. Louis, MO) and 7.5 μL ethylenediamine acetate buffer 0.05 M (pH 7.0), the second sample in 20 μL *Flavobacterium heparinum* extract and the third sample in 20 μL MilliQ water. The samples were incubated overnight at 30°C, lyophilized, suspended in 5 μL MilliQ water, and analyzed by agarose gel electrophoresis as described above. The crude extract of *Flavobacterium heparinum* grown in the presence of heparin degrades heparin and HS [35]. Heparitinases I and II, and a heparin lyase have been identified in the crude extract of *Flavobacterium heparinum* [36,37]. The crude extract of *Flavobacterium heparinum* also degrades HA, C4S and C6S [38]. In addition, the crude extract of *Flavobacterium heparinum* grown in the presence of CS degrades DS [39,40].

Digestion control samples (DS, C4S, HS, and a mixture of CS, DS and HS extracted from shark cartilage; all 1 mg/mL) were also incubated in *Flavobacterium heparinum* extract, heparin lyase II or MilliQ water overnight at 30°C and analyzed by agarose gel electrophoresis.

### Quantification of hyaluronic acid

HA was quantified as previously described [41]. Briefly, 96-well plates were incubated with purified HA binding protein extracted from bovine nasal cartilage in coating buffer (0.06 M NaHCO_3_, 0.5 g/L sodium azide, pH 9.6) (100 μL/well) overnight at 4°C. The plates were then washed three times with washing buffer (0.05 M Tris—HCl, 0.15 M NaCl, 0.05% Tween 20, 0.02 mM EDTA III, 7.7 mM sodium azide, pH 7.75). Blocking buffer (the washing buffer containing 1% bovine serum albumin (BSA)) was added to each well (200 μL/well) and the plates kept at 4°C for at least 2 hours, the blocking buffer being removed only when the samples were added. GAGs (20 μg) extracted from archaeological human bone samples were suspended in blocking buffer, and 100 μL added to each well (triplicate). Standard HA (extracted from human umbilical cord; Sigma-Aldrich) prepared in blocking buffer was added to the plate (100 μL/well, triplicate) to produce a concentration curve (0, 0.48, 1.95, 7.8, 31.2, 125, 500 and 1000 ng/mL HA). The plates were kept overnight at 4°C. The plates were then washed 5 times with washing buffer, biotinylated HA binding protein diluted 1:5000 in blocking buffer was added (100 μL/well) and the plates kept at room temperature for 2 hours on a Delfia PlateShake (1296–004; Perkin Elmer, Turku, Finland). The plates were then washed 5 times in washing buffer. Europium-conjugated streptavidin (Delfia Eu-labelling kit, Perkin Elmer) diluted 1:10,000 in blocking buffer was added (100 μL/well) and the plates kept at room temperature for 30 min on the shaker. The plates were then washed 5 times with washing buffer and incubated in Delfia Enhancement solution (200 μL/well) (Perkin Elmer) for 5 min at room temperature on the shaker. The Enhancement solution was added using a Delfia Plate Dispenser (1296–041; Perkin Elmer). The plate was read using an Elisa ELX 800 Wallac Victor^2^ 1420 Multilabel Counter (Perkin Elmer). The quantity of HA was then calculated per mg of protein.

### Quantification of proteins

The quantity of proteins extracted (i.e. from 100 mg of bone powder and 25 mg of tooth powder) was calculated using a Pierce BCA Protein Assay Kit (Thermo Scientific, Rockford, IL) according to the manufacturer’s protocol.

### Immunohistochemistry

Transverse bone sections (60 μm thick) were obtained using a Leica SP1600 Saw Microtome (Leica Biosystems, Nussloch, Germany). Bone decalcification results in loss of proteins, and by using the Leica SP1600 Saw Microtome, which is comprised of a diamond-coated blade, hard materials such as bone can be sliced without any previous treatment that could lead to changes in bone composition and structure. The bone slices were hydrated in PBS buffer for 1 hour at 4°C and then fixed in 2% buffered paraformaldehyde for 30 min followed by washing in PBS and antigen recovery (10 min incubation in 10 mM sodium citrate pH 6 at 100°C). The bone slices were then washed, incubated in 10% hydrogen peroxide for 30 min, washed, and unspecific protein binding sites were blocked with 5% fetal bovine serum (FBS). The slices were then incubated with primary antibodies, or biotinylated HA binding protein (prepared in the laboratory [42]), overnight at 4°C. The primary antibodies used were: rabbit anti-fibromodulin (H-50, Santa Cruz, Santa Cruz, CA), goat anti-decorin (22613, Santa Cruz), rabbit anti-biglycan (H-150, Santa Cruz), goat anti-glypican 1 (14645, Santa Cruz), mouse anti-chondroitin 6-sulfate (Millipore MAB2035), mouse anti-bone sialoprotein (BSP) II (73497, Santa Cruz), rabbit anti-osteocalcin (30044, Santa Cruz) and goat anti-collagen IAI (25974 Santa Cruz). Prior to using the antibody mouse anti-chondroitin 6-sulfate (Millipore MAB2035), the bone sections were incubated in chondroitin AC lyase, overnight at 37°C. Bone sections were washed and then processed using the Universal Dako LSAB+ Kit, Peroxidase (LSAB+ Kit, HRP) and Dako Liquid DAB+ Substrate Chromogen System (Dako, Glostrup, Denmark) (incubation with the biotinylated link from the LSAB+ Kit was omitted when the biotinylated HA binding protein was used). Finally, bone sections were incubated sequentially in 30% ethanol, 50% ethanol, 70% ethanol, 90% ethanol, 100% ethanol, ethanol:xylol (1:1), xylol, and then mounted on glass slides in Permount (Thermo Fisher Scientific Inc., Waltham, MA) and sealed with nail polish. Negative control immunostainings were performed with the omission of each primary antibody, in the presence of FBS, overnight at 4°C.

### HPLC

The disaccharide sulfation pattern of the CS population extracted from archaeological human adolescent femur was determined by strong anion exchange high pressure liquid chromatography (HPLC). The chromatographic equipment included a U3000HPLC from Dionex, two single piston pumps, a RF2000 fluorescence detector from Dionex with a 12-μl flow cell volume, a dry reaction bath (FH-40) and a thermocontroller (TC-55) from Brinkman Instruments as previously described [43]. The disaccharides were separated using a Dionex Sax column (2.4 mm x 150 mm). The flow rate was 0.5 ml/min and the gradient was performed using Pump 1 at 50% of the total flow rate with Buffer A, 0.00316 M HCL pH 2.5 and Buffer B, 1 M NaCl. Subsequently, 1.0% NaOH and 0.5% 2-cyanoacetamide at a proportion of 1:1 was mixed to the effluent using Pump 2 at 50% of the flow rate. The mixture was passed through a reaction coil (0.25 μm inner diameter 15 m long) set in a dry reaction temperature controlled chamber set at 125°C and monitored fluorometrically (excitation wavelength, 346 nm; emission wavelength, 410 nm). Peaks were identified based on the retention times of known CS standards: C6S was isolated from shark cartilage (Sigma) and the standard C4S:C6S (~30:70%) was isolated from chick embryo epiphyseal cartilage. The disaccharides were generated by previously digesting the samples with Chondroitinase AC. The retention times were compared to previously characterized profiles [44].

## Results

### Glycosaminoglycans extracted from archaeological human bones and teeth

**Fig 1:**
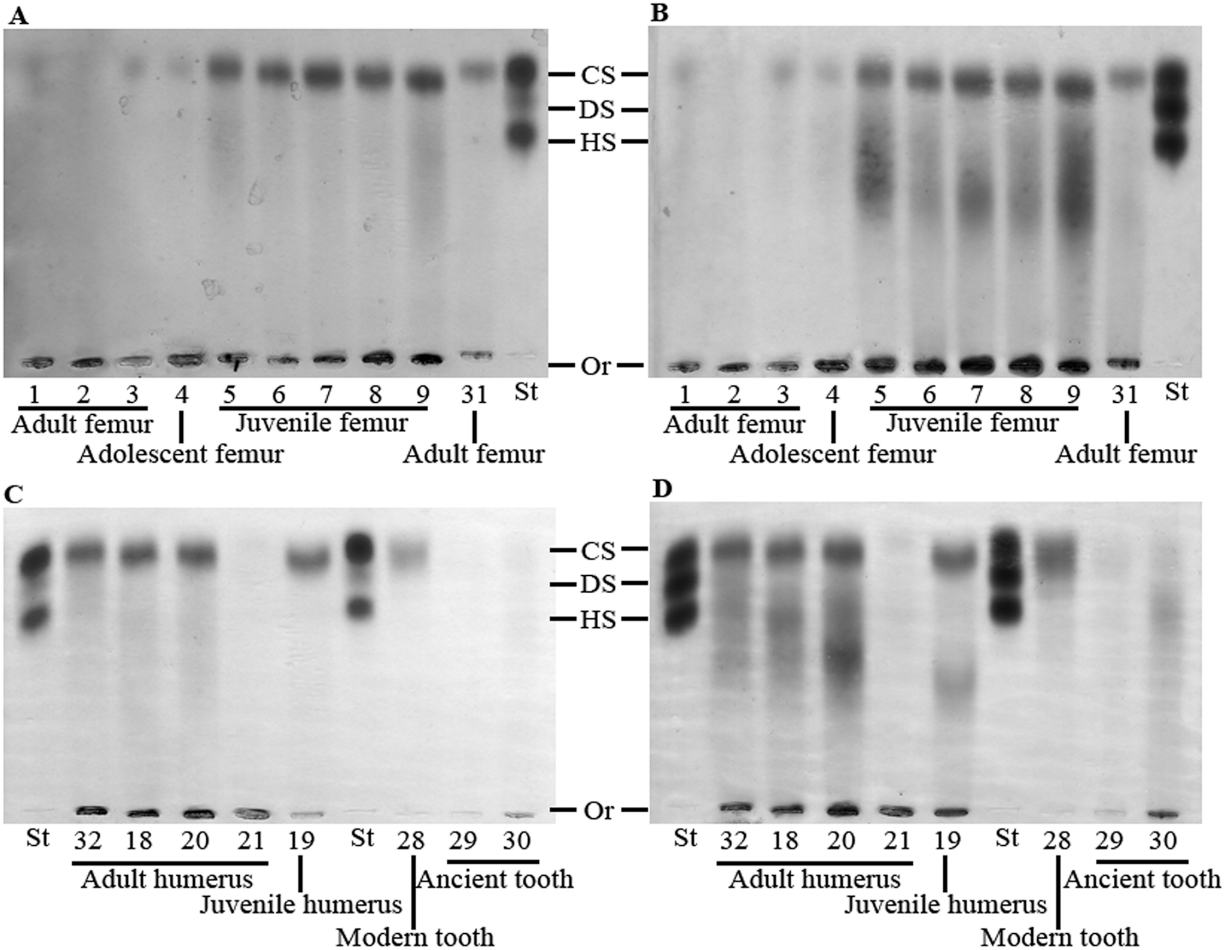
Electrophoresis in PDA buffer of glycosaminoglycans extracted from archaeological human bones and teeth. (A and C) GAGs isolated from archaeological human bones and teeth, as well as from a modern adult human tooth, were analyzed by agarose gel electrophoresis in PDA buffer. The gels were stained with 0.1% toluidine blue prepared in a solution containing 1% acetic acid and 50% ethanol, and destained with the same solution without toluidine blue (for staining CS, DS and HS). (B and D) After photography of (A), the gels were stained with 0.1% toluidine blue prepared in 25 mM sodium acetate buffer, pH 5.0, and destained in this solution without toluidine blue (for also staining HA). CS: chondroitin sulfate; DS: dermatan sulfate; HS: heparan sulfate; St: standard (CS, DS and HS extracted from shark cartilage); Or: origin.

**Fig 2:**
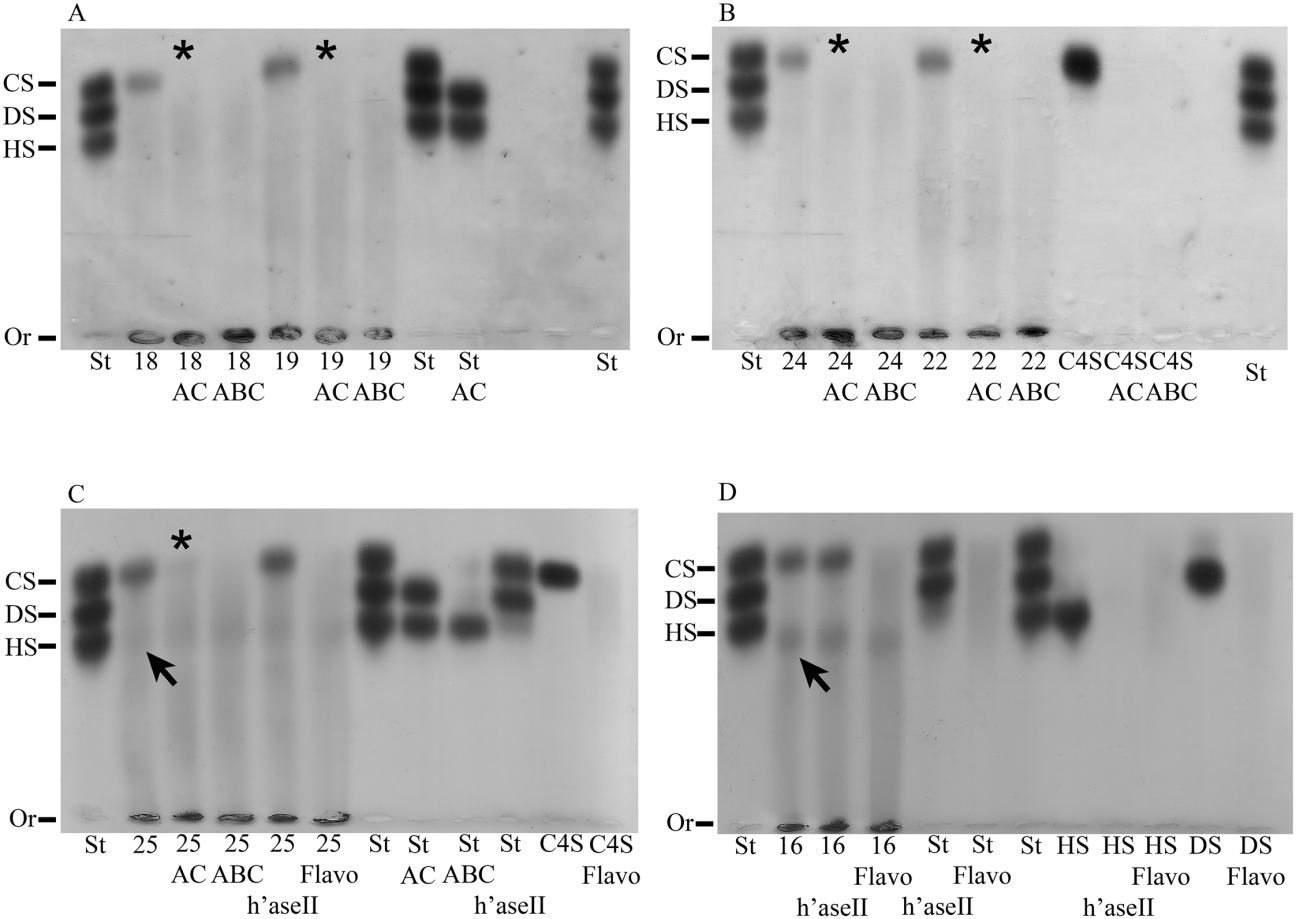
Electrophoresis in PDA buffer of glycosaminoglycans extracted from archaeological bones and degraded with glycosaminoglycan-specific enzymes. GAGs were extracted from archaeological human adult and juvenile humerus (A), archaeological human adult and adolescent ulna (B and C), and archaeological human adult radius (D), subjected to β-elimination in some instances (samples 16 and 25), digested with chondroitin AC lyase, chondroitin ABC lyase, heparin lyase II or *Flavobacterium heparinum* extract, and analyzed by agarose gel electrophoresis in PDA buffer. The gels were stained with 0.1% toluidine blue prepared in a solution containing 1% acetic acid and 50% ethanol, destained with the same solution without toluidine blue, restained with 0.1% toluidine blue prepared in 25 mM sodium acetate buffer, pH 5.0, and destained in this solution without toluidine blue. CS: chondroitin sulfate; DS: dermatan sulfate; HS: heparan sulfate; Or: origin; St: standard (CS, DS and HS extracted from shark cartilage); C4S: chondroitin 4-sulfate; AC: chondroitin AC lyase; ABC: chondroitin ABC lyase; h’aseII: heparin lyase II; Flavo: *Flavobacterium heparinum* extract; 16: adult radius; 18: adult humerus; 19: juvenile humerus; 22: adolescent ulna; 24: adult ulna; 25: adult ulna.

GAGs were extracted from archaeological human bones and teeth and analyzed by agarose gel electrophoresis in PDA buffer, whereby GAGs are separated according to their affinity with the buffer and can be visualized by toluidine blue staining co-migrating with the standard control (composed of CS, DS and HS). GAGs extracted from a modern wisdom tooth served as a comparative positive control. The electrophoretic profile of the GAGs isolated from bone and tooth samples revealed the presence of a CS population for all samples, detected as a single band in the gels (Fig 1A and 1C). This CS population was confirmed to be CS chains by treatment with specific glycosidases as discussed below. When the same agarose gels were restained and destained with sodium acetate buffer, HA was also revealed for all samples, detected as a streak in the gels (Fig 1B and 1D). The GAG profiles were similar for all samples; however, a third GAG population that co-migrated with HS was also detected in two samples; adult radius and adult ulna (arrows in Fig 2).

The densitometric measurements of the CS band are shown in Table 1. As can be observed, the total quantity of CS extracted from 100 mg of bone or tooth powder varied between 1.51 and 23.36 μg, with a mean quantity of 10.3 μg. The CS yield for some bone samples was higher than for others; more CS was extracted from juvenile femur than from adult and adolescent femur, although this was not the case for humeri and tibiae, where similar amounts were extracted from adult and juvenile samples, and the opposite was seen for ulna samples. However, GAGs could be isolated from all samples analyzed and clear profiles observed even when only small sample sizes were available, and there was no indication that preference should be given to one bone over another when extracting GAGs from archaeological samples. Approximately one fifth of the quantity of CS extracted from the modern tooth was extracted from the two archaeological teeth thereby indicating that at least 20% of the GAGs were preserved in ancient teeth. These GAGs could, however, be in the form of intact GAG chains or most likely GAG fragments of a size greater than a tetrasaccharide since smaller fragments are not observed by agarose gel electrophoresis in PDA buffer.

**Table 1:** Densitometric quantification of chondroitin sulfate extracted from archaeological human bones and teeth.

Type of bone	ID No.	CS (μg)^a^	CS in 100 mg of mineralized tissue (μg)^b^	Protein in 100 mg of mineralized tissue (mg)^c^	CS in μg/mg of protein^d^
Adult femur	1	0.959	8.630	0.512	16.855
	2	0.343	3.091	0.429	7.205
	3	1.072	11.153	0.397	28.093
	31	1.624	6.496	0.404	16.079
Adolescent femur	4	0.781	7.184	0.344	20.884
Juvenile femur	5	2.211	8.845	0.356	24.845
	6	2.346	9.385	0.165	56.878
	7	3.046	12.186	0.236	51.636
	8	2.769	11.075	0.227	48.789
	9	3.466	20.797	0.217	95.838
Adult tibia	10	0.973	15.564	0.396	39.303
	11	3.540	14.159	0.224	63.210
	12	0.721	6.631	0.655	10.124
	13	2.433	19.460	0.364	53.462
Juvenile tibia	14	2.850	11.400	0.195	58.462
	15	3.069	12.274	0.228	53.833
Adult radius	16	2.467	9.867	0.372	26.524
	17	2.010	8.039	0.450	17.864
Adult ulna	23	2.152	8.606	0.231	37.255
	24	3.062	6.125	0.223	27.466
	25	3.782	15.129	0.205	73.800
Adolescent ulna	22	3.096	6.193	0.180	34.406
Juvenile ulna	26	0.377	1.508	0.186	8.108
	27	1.157	4.627	0.258	17.934
Adult humerus	18	3.326	17.297	0.243	71.181
	20	3.913	15.651	0.270	57.967
	21	0.453	3.622	0.425	8.522
	32	3.388	13.552	0.492	27.545
Juvenile humerus	19	3.773	7.545	0.293	25.751
Modern tooth	28	4.380	23.358	1.366	17.100
Ancient tooth	29	0.578	4.622	0.670	6.899
	30	0.624	6.242	1.050	5.945

^a^ The CS band observed in the gels in Fig 1 was quantified by densitometry in comparison to the standard CS band (or average value for gels with more than one standard).

^b^ The total quantity of CS extracted from 100 mg of bone or tooth powder was calculated.

^c^ The total quantity of protein extracted from 100 mg of bone or tooth powder was calculated.

^d^ The total quantity of CS was divided by the total quantity of protein extracted from 100 mg of bone or tooth powder.

In order to investigate whether the smear revealed in the agarose gel electrophoresis, when distained with sodium acetate, was indeed HA (Fig 1), the HA content of ten samples was quantified using a previously described probe-based sandwich ELISA assay [41]. A significant quantity of HA was detected in all samples analyzed (Table 2).

**Table 2:** Quantification of hyaluronic acid extracted from archaeological human bones and teeth by probe-based sandwich ELISA assay.

Type of bone	ID No.	HA in 100 mg of mineralized tissue (ng)^a^	Protein in 100 mg of mineralized tissue (mg)^b^	HA in ng/mg of protein ^c^
Adult femur	31	3.982	0.215	18.521
Adolescent femur	4	1.119	0.350	3.197
Juvenile femur	8	0.843	0.323	2.610
	9	0.795	0.710	1.120
Adult radius	16	0.965	0.185	5.216
Adult humerus	18	0.598	0.305	1.961
Juvenile humerus	19	0.559	0.346	1.616
Adolescent ulna	22	0.354	0.180	1.967
Adult ulna	24	0.634	0.547	1.159
	25	5.169	0.509	10.155

^a^ The total quantity of HA extracted from 100 mg of bone powder was calculated.

^b^ The total quantity of protein extracted from 100 mg of bone powder was calculated.

^c^ The total quantity of HA was divided by the total quantity of protein extracted from 100 mg of bone or tooth powder.

As mentioned above, the GAGs extracted from nine samples were degraded with chondroitin AC lyase or chondroitin ABC lyase, specific glycosidases, in order to identify whether the electrophoretic band observed in Fig 1 was solely CS or a mixture of CS and DS. Chondroitin AC lyase degrades polysaccharide chains containing β-(1–4) and β-(1–3) linkages between hexosamines and glucuronic acid residues to oligosaccharides, mainly disaccharides, and therefore degrades CS, whilst chondroitin ABC lyase degrades polysaccharides containing β-(1–4)-D-hexosaminyl and β-(1–3)-D-glucuronosyl or α-(1–3)-L-iduronosyl linkages to disaccharides and therefore degrades both CS and DS. The GAGs were analyzed by agarose gel electrophoresis in PDA buffer following digestion. Small GAG fragments, such as degraded GAG fragments, are not precipitated by CTV and therefore do not appear in stained gels. The electrophoretic band disappeared from the gels following digestion with chondroitinases AC and ABC in all instances (five of the samples are shown in Fig 2). Since the electrophoretic band disappeared following digestion with chondroitin AC lyase, which degrades CS but not DS, the isolated CS population consisted solely of CS (asterisks in Fig 2).

In the case of two archaeological human bone samples, an electrophoretic band migrating in line with HS standard was also observed (arrows in Fig 2). GAGs extracted from these two samples were digested with heparin lyase II (which degrades HS) or *Flavobacterium heparinum* extract (which degrades CS, DS and HS) in order to confirm whether this band was a HS population. The degraded GAGs were analyzed by agarose gel electrophoresis in PDA buffer. All positive controls indicated that the enzymes were working well (Fig 2). The electrophoretic band migrating in line with HS was not susceptible to digestion with heparin lyase II or *Flavobacterium heparinum* extract. Therefore, this fragment could reveal valuable information and is currently being analyzed; however, it is beyond the scope of this study (Fig 2).

### HPLC profile of chondroitin sulfate isolated from archaeological human bones

**Fig 3:**
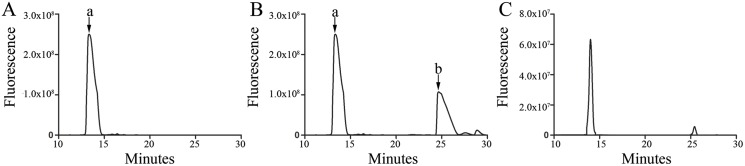
Disaccharide content of the CS population extracted from archaeological human adolescent femur was characterized by strong anion exchange liquid chromatography. (A) C6S standard profile; (B) C4S/C6S Standard profile; and (C) profile of CS extracted from archaeological human adolescent femur. (a) Peak representing the C6S disaccharide; and (b) peak representing the C4S disaccharide.

The CS population isolated from two of the archaeological human bones was analyzed by strong anion exchange using HPLC to determine the sulfation pattern of CS. The chromatogram showed a large C6S peak comprising over 95% of the CS (Fig 3C). A smaller C4S peak was also observed comprising less than 5% of the CS (Fig 3C). Therefore, this data reveals it is possible to characterize the fine structure of the GAGs purified from archaeological bones.

### Immunolocalization of glycosaminoglycans in archaeological bones

**Fig 4:**
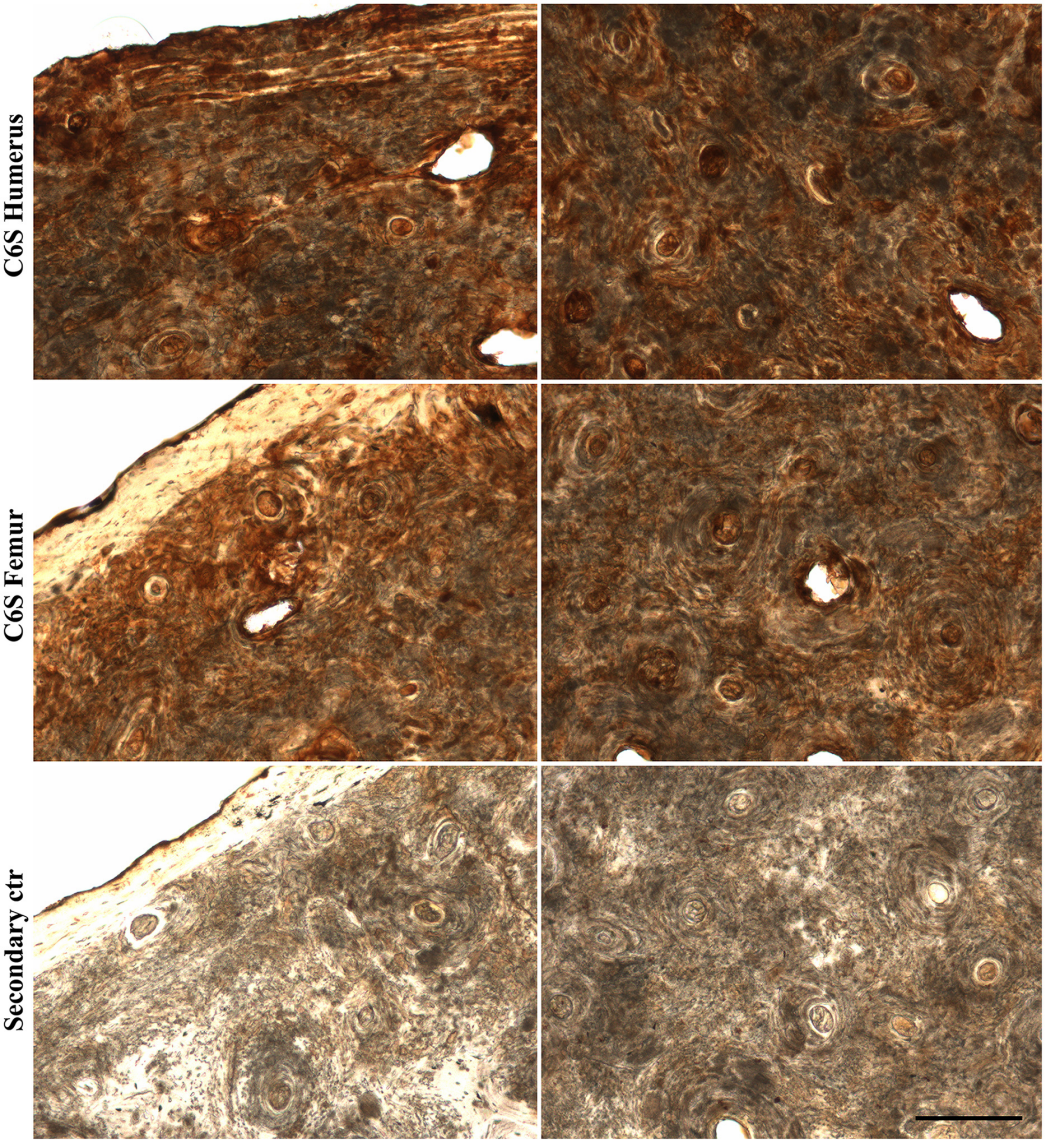
Immunolocalization of chondroitin-6-sulfate in archaeological human bone slices. Archaeological human adult humerus or femur slices (60 μm thick) were labeled with anti-chondroitin-6-sulfate (following digestion with chondroitin AC lyase) and developed using DAB (brown). The secondary control was prepared omitting the primary antibody (archaeological human adult femur is shown). Cortical bone is viewed on the left and trabecular bone on the right. Scale bar: 200 μm.

**Fig 5:**
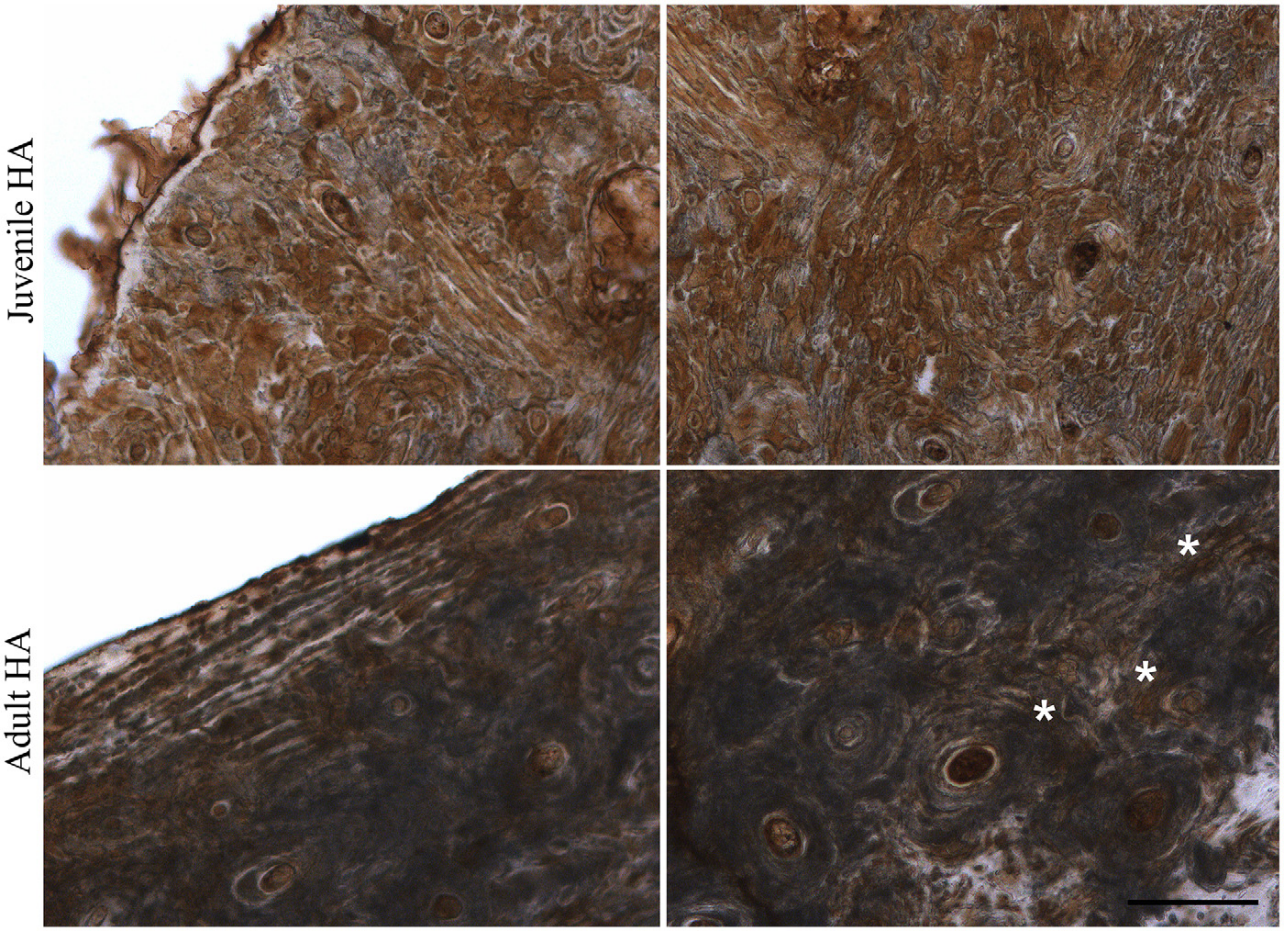
Immunolocalization of hyaluronic acid in archaeological human bone slices. Archaeological human adult or juvenile humerus slices (60 μm thick) were labeled with biotinylated binding protein and developed using DAB (brown). Cortical bone is viewed on the left and trabecular bone on the right. The asterisks show HA in a concentric pattern surrounding the osteons. Scale bar: 200 μm.

The GAG groups isolated from all archaeological human bone and tooth samples were CS and HA, and HPLC analysis showed C6S to be the major type of CS. We analyzed the location of C6S and HA in archaeological human bone slices. Due to the potential fragility and already compromised structure of the archaeological bones, care was taken to obtain bone slices with no previous decalcification. C6S was immunostained using anti-chondroitin 6-sulfate (MAB2035, Millipore) following digestion with chondroitin AC lyase since this antibody recognizes C6S stubs, and HA was labeled using biotinylated HA binding protein. C6S was analyzed in adult humerus and adult femur. C6S staining was similar for both types of bone; evenly distributed throughout the bone matrix (Fig 4). HA was analyzed in adult humerus and juvenile humerus. A different immunostaining pattern was observed for the two types of bone; more intense and evenly distributed throughout the bone matrix in juvenile bone slices (Fig 5), and in a concentric pattern surrounding the osteons in adult bone slices (asterisks in Fig 5).

### Proteoglycans extracted from archaeological human bones and teeth

**Fig 6:**
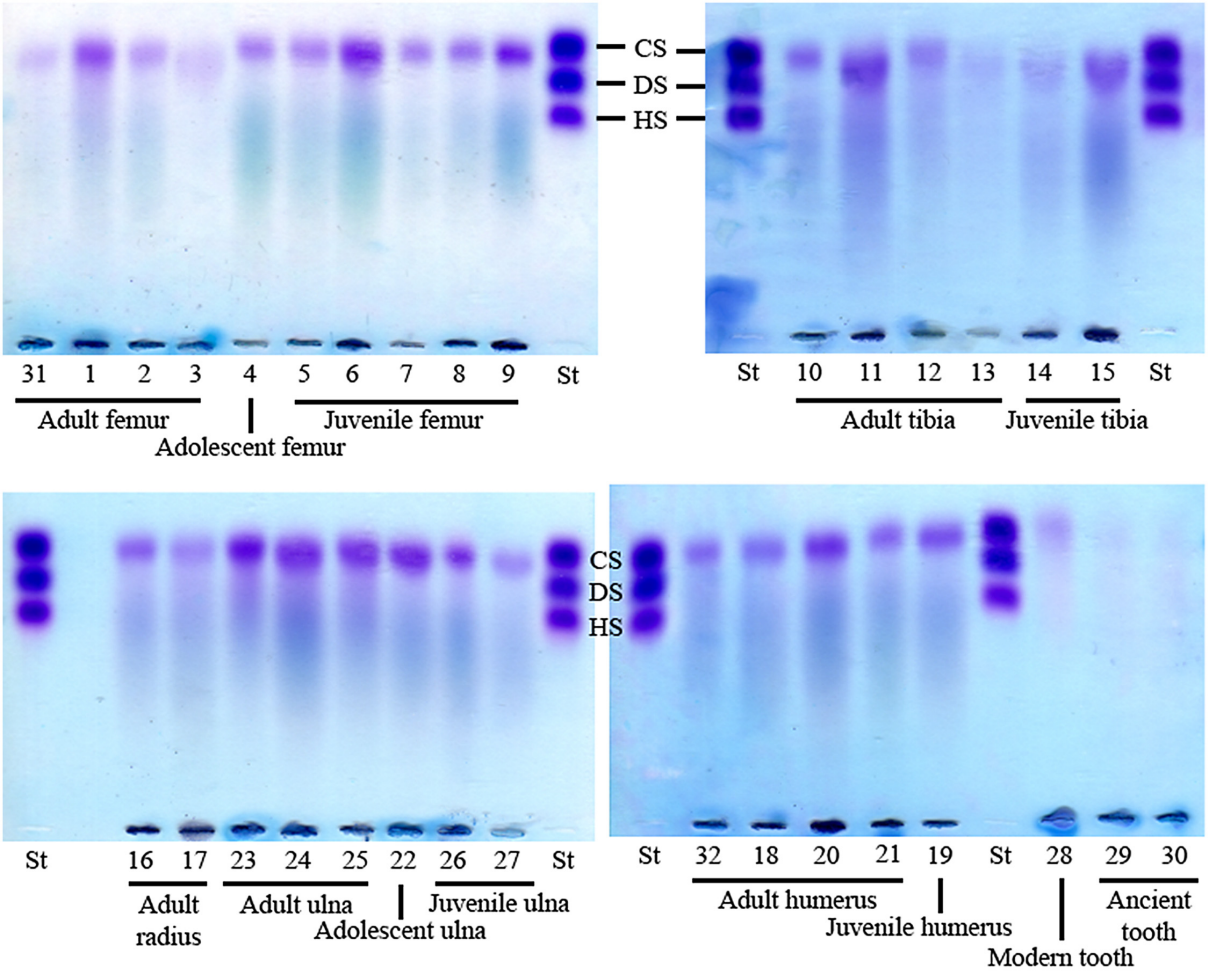
Proteoglycans extracted from ancient bones and teeth. PGs were extracted from various types of archaeological human bone samples (femur, humerus, radius, tibia and ulna) identified as juvenile, adolescent or adult, and also archaeological adult teeth and a modern wisdom tooth. Extracted PGs were analyzed by agarose gel electrophoresis in PDA buffer, and stained by amide black followed by toluidine blue. Proteins are stained in blue and GAG chains in purple. CS: chondroitin sulfate; DS: dermatan sulfate; HS: heparan sulfate; St: standard (CS, DS and HS extracted from shark cartilage).

PGs were successfully extracted from all of the archaeological human bone and tooth samples analyzed, and the PG profile was compared to that of a modern wisdom tooth. The PGs were analyzed by agarose gel electrophoresis in PDA buffer, and a PG band was observed for all samples co-migrating with the GAG standard CS (Fig 6). Since the GAG analysis discussed above detected CS but not DS in the archaeological human bones and teeth, this PG population is a CSPG and not a CS/DSPG. The PG profile of archaeological bones was similar to that of a modern tooth (Fig 6). Proteins co-extracted with the PGs were observed in the gel as a smear by amide black staining (stained in blue in Fig 6) followed by the toluidine blue staining for PGs (stained in purple in Fig 6).

### Immunolocalization of proteoglycans in archaeological human bones

**Fig 7:**
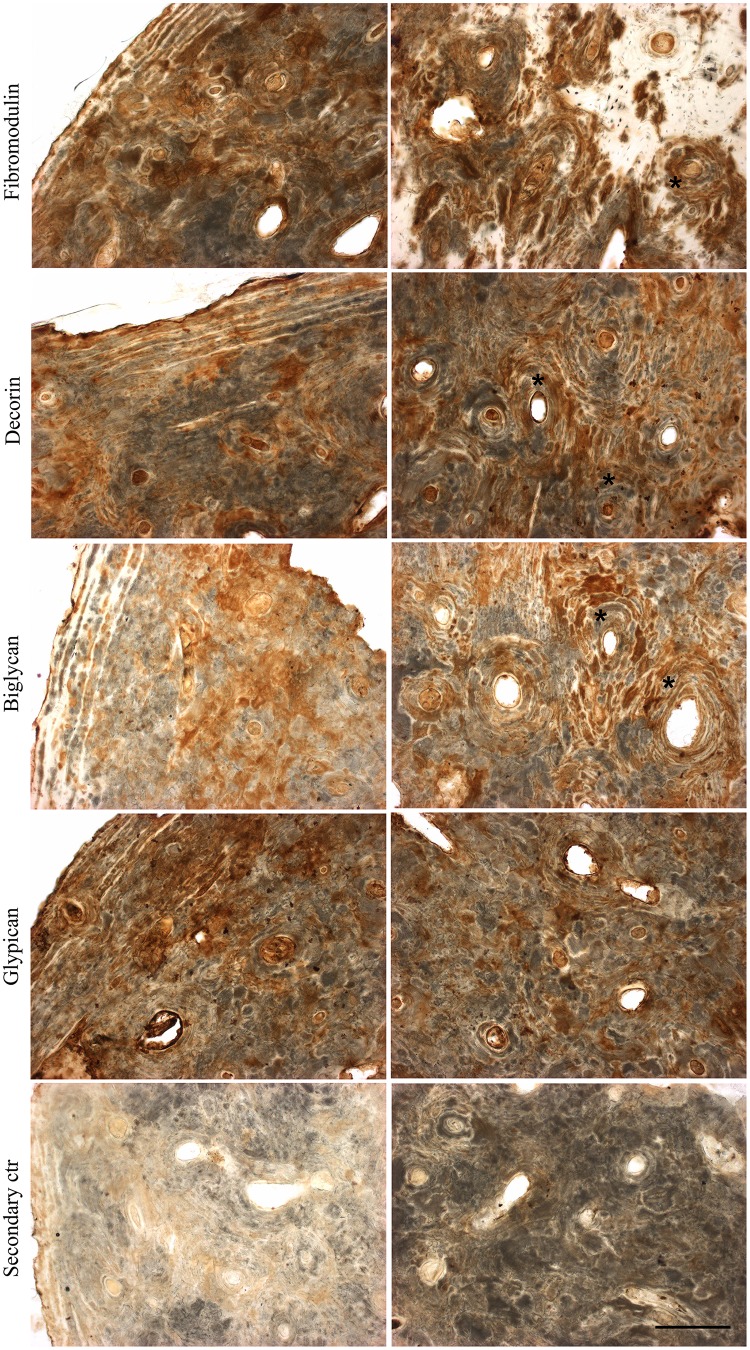
Immunolocalization of proteoglycans in archaeological human adult bone slices. Archaeological human adult humerus slices (60 μm thick) were labeled for decorin, biglycan, fibromodulin or glypican, and developed using DAB (brown). The secondary control was prepared omitting the primary antibodies. Cortical bone is viewed on the left and trabecular bone on the right. Scale bar: 200 μm.

**Fig 8:**
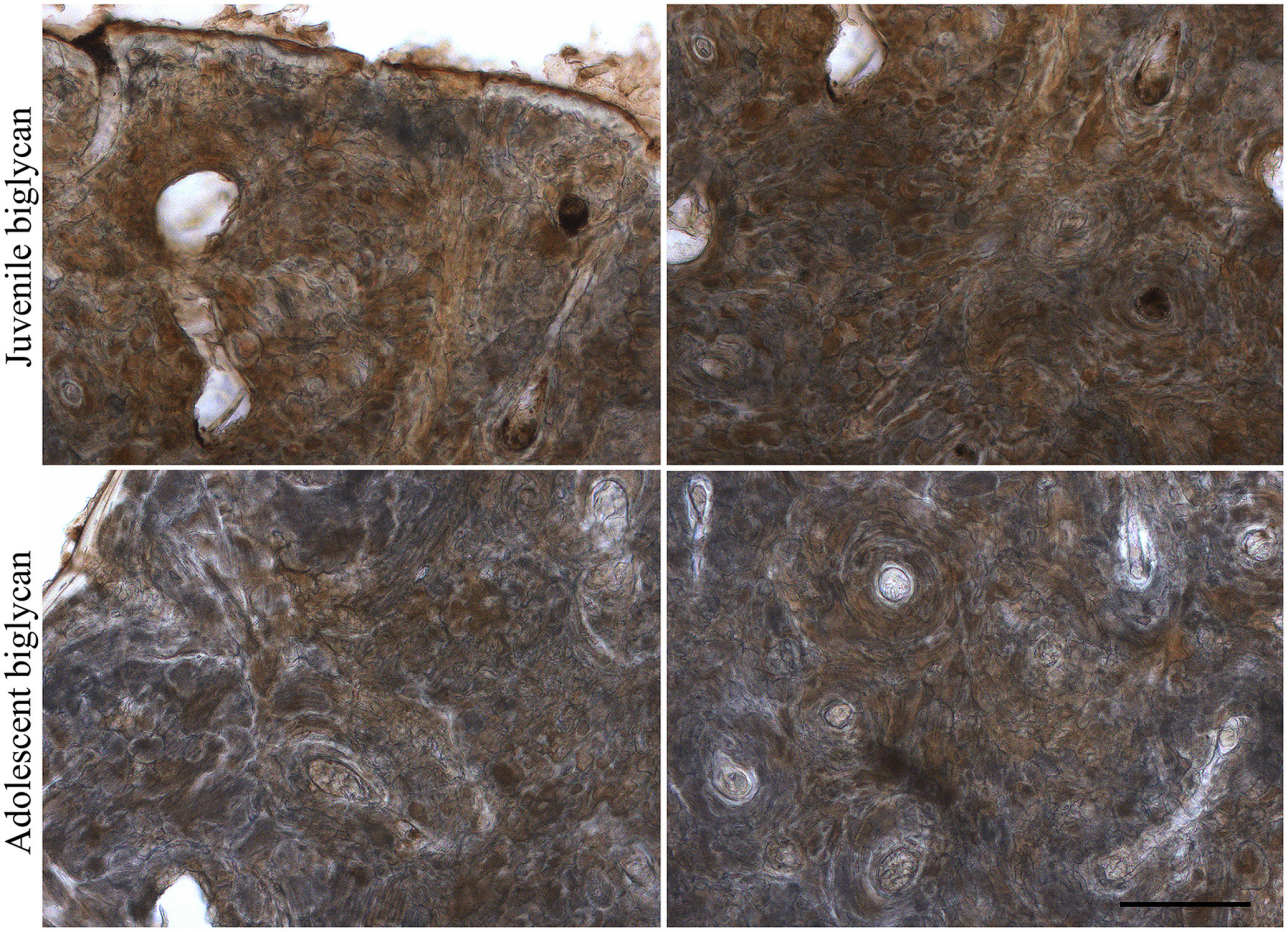
Immunolocalization of biglycan in archaeological human juvenile and adolescent bone slices. Archaeological human juvenile humerus and adolescent femur slices (70 μm thick) were labeled for biglycan and developed using DAB (brown). Cortical bone is viewed on the left and trabecular bone on the right. Scale bar: 200 μm.

For a more in-depth analysis as to which PG populations are present in archaeological human bones, immunohistochemistry was performed on archaeological human adult bone slices, particularly for SLRPs, which are an important family of PGs described in bones playing a major role during all phases of bone formation. Fibromodulin, decorin, biglycan and glypican were detected in the archaeological human bone slices, and each PG presented a distinct distribution pattern throughout the bone tissue (Fig 7). As can be seen in Fig 7, fibromodulin, decorin and biglycan appear to be more closely associated with osteons in trabecular bone tissue (indicated with asterisks) and diffused in cortical bone tissue, whilst glypican is diffused throughout the bone matrix in both types of bone tissue. There is less intense staining for glypican compared to the other three PGs (Fig 7). In juvenile bones, biglycan was observed evenly distributed throughout the bone matrix, whilst in adolescent bones it had a similar distribution pattern to adult bones (Fig 8).

**Fig 9:**
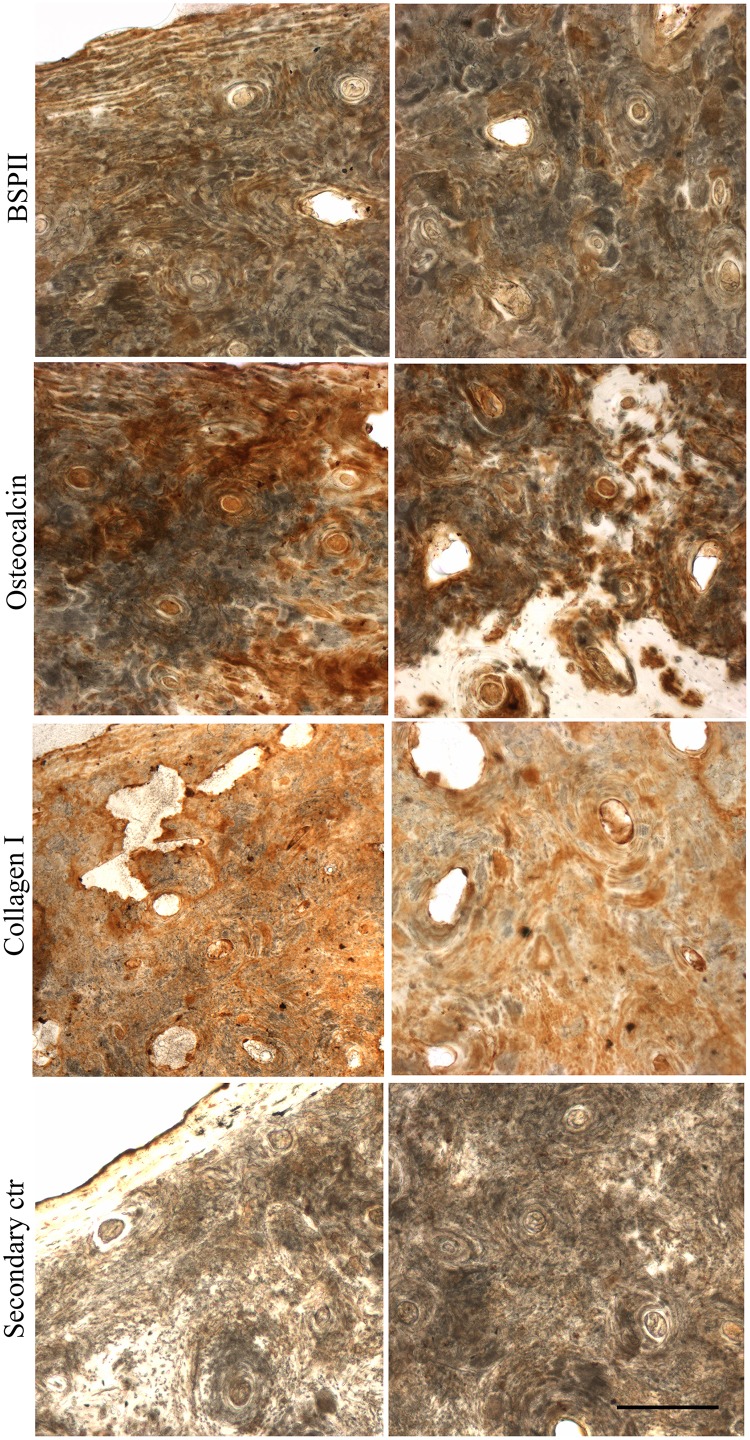
Immunolocalization of BSPII, osteocalcin and collagen I in archaeological human bone slices. Archaeological human femur slices (60 μm thick) were labeled for BSPII, osteocalcin and collagen I, and developed using DAB (brown). Cortical bone is viewed on the left and trabecular bone on the right. Scale bar: 200 μm.

The localization of collagen types I and III and the non-collagenous proteins osteocalcin and bone sialoprotein II was also analyzed by immunohistochemistry since these proteins are present in a substantial amount in organic bone matrix. As can be seen in Fig 9, collagen I was evenly distributed throughout the bone tissue, whilst osteocalcin and bone sialoprotein II had an irregular distribution pattern.

## Discussion

For the first time, PGs and GAGs were isolated from archaeological human bones and teeth. PGs and their constituent GAG chains bind to hydroxyapatite [4,5], which would protect these GAGs from degradation over the years. HS, heparin and DS have been shown to bind more strongly to hydroxyapatite than C6S, C4S and HA [45]. However, our research showed the presence of C6S, C4S and HA, but not of HS or DS, in archaeological human bone samples, and detected only the protein core of a HSPG and a KSPG, suggesting that other factors could play a role in GAG preservation. Moreover, some PGs and GAGs in archaeological bones and teeth could be degraded more quickly than others due to extrinsic factors, such as enzymes produced by bacteria in the environment [46,47], and intrinsic factors, such as binding to collagen [48,49,50].

The GAGs could be identified by electrophoretic mobility in 1,3-diaminopropane with the use of buffers at different pHs and by digestion with specific enzymes. The GAGs observed for all bone and tooth samples were CS and HA. In addition, an unidentified electrophoretic band was also observed for two archaeological human bone samples. Our results are similar to those of a study of GAGs from human femoral compact bone in autopsy cases and of a study of GAGs from human dentine and cementum of adult patients, which describe CS and HA; CS as the predominant GAG and HA as a minor proportion of the GAGs [51,52]. Previous research of human alveolar bone samples obtained from oral surgical procedures has identified CS, DS, HS and HA in non-demineralized extracts and only CS in demineralized samples [53]. DS and HS were not detected in the present study, even though the samples had not been demineralized, but alveolar bone was not amongst the types of bone analyzed, which included femur, humerus, tibia, radius and ulna.

The quantities of CS and HA extracted did not appear to be dependent on the type of bone or whether it was a juvenile, adolescent or adult bone, but most likely on how degraded the bone was, although the only bone samples that stood out as being more degraded than the others were sample ID nos. 16 and 25 (adult radius and adult ulna, respectively), which crumbled when sliced using the microtome, but this could be due to other reasons such as disease. Interestingly, these were the two bone samples for which an unidentified electrophoretic band was observed.

HPLC analysis identified the CS population extracted from the archaeological human bone samples as over 95% C6S and less than 5% C4S. Previous research has reported a CS population of predominantly C6S in a mixture of human dentine and cementum from fresh adult teeth, with smaller amounts of C4S [51]. Our findings differ to those observed for human femoral compact bone in autopsy cases and human alveolar bone samples obtained from oral surgical procedures, which identified only C4S as the extracted CS [52,53].

The localization of C6S and HA was analyzed in archaeological human bone slices. C6S was analyzed in two types of archaeological human bone; adult humerus and adult femur. The staining pattern for both types of bone was similar; evenly distributed throughout the bone matrix. HA was analyzed in adult humerus and juvenile humerus, and a different staining pattern was observed; distributed throughout the bone matrix in juvenile bone and in a concentric pattern around the osteons in adult bone. The different distribution patterns of HA in juvenile and adult bones could reflect the role of this GAG in bones, which includes bone resorption, osteoclast motility and osteoblast differentiation [54,55,56].

A CSPG population was identified in both archaeological human bones and teeth. CS has been shown to be associated with a protein moiety in human dentine from freshly extracted teeth [57]. Immunohistochemistry assays showed positive labeling for two CS/DS SLRPs, biglycan and decorin. Biglycan and decorin bind to hydroxyapatite in bone [58], which would have protected these molecules from degradation over the years. A study of biglycan and decorin expression in rat cell culture models has shown that these two SLRPs contain mainly DS substitutions during early bone matrix formation, and carry only CS chains during mineralization [12], which could explain why CS but not DS was extracted from the archaeological human bone samples. A study of adult human bone biopsies has shown that decorin is observed mostly in the perilacunar matrix, canaliculi of osteocytes, and matrix immediately adjacent to quiescent Haversian canals, whilst biglycan is evenly distributed throughout cortical and trabecular bone matrix [59]. In our study, biglycan and decorin appeared to be closely associated with osteons in adult archaeological human bones. In juvenile bones, biglycan was observed evenly distributed throughout the bone matrix, whilst in adolescent bones it had a similar distribution pattern to adult bones.

Immunohistochemistry results also showed positive labeling for a cell surface HSPG, glypican-1, although there was less staining for glypican-1 than for the two SLRPs, decorin and biglycan. However, the GAGs isolated from the archaeological human bone samples did not include HS. The glypican-1 antibody used binds to the protein core and therefore the protein core would have been detected in the immunohistochemistry assays. In intact cells, glypican is linked to the cell membrane by glycosylphosphatidylinositol linkages; however, considering that cells are no longer present in archaeological bones, the glypican-1 protein core, or fragments thereof, would have become attached to the ECM during cell death. Fibromodulin, a SLRP with KS substitutions, was also detected by immunohistochemistry. The fibromodulin antibody used binds to the protein core, and therefore the protein core would have been detected in the immunohistochemistry assays. KS was not detected in our study but has previously been described in human bone [60]. Furthermore, the unidentified electrophoretic band migrating in line with HS which was not susceptible to digestion with heparin lyase II or *Flavobacterium heparinum* extract could still prove to be KS in future experiments.

Collagen type I is the most abundant collagen type in bone. Together with several non-collagenous proteins, such as osteocalcin and osteopontin, collagen I forms a scaffold for mineral deposition, and SLRPs regulate collagen fibril assembly and diameter [2,61]. Immunohistochemistry results showed collagen I evenly distributed throughout the bone tissue in archaeological human bones. Osteocalcin and bone sialoprotein II, which are specific to bone, were also observed in the archaeological bone samples, with an irregular distribution pattern.

## Conclusions

This is the first time to our knowledge that PGs and GAGs have been analyzed in archaeological human bones and teeth. PGs and GAGs play a major role in bone morphogenesis, homeostasis and degenerative bone disease, and the ability to isolate and characterize PG and GAG content from archaeological skeletons could unveil valuable information. Our methods enable the extraction and analysis of GAGs from small amounts of archaeological human bone and tooth samples, and in this study, clear profiles were observed for all samples analyzed. The finding that significant quantities of PGs and GAGs persist in archaeological bones and teeth opens novel venues for the fields of Paleontology and Forensic Science.
